# The Great Mime: Three Cases of Melanoma with Carcinoid-Like and Paraganglioma-Like Pattern with Emphasis on Differential Diagnosis

**DOI:** 10.3390/dermatopathology8020019

**Published:** 2021-05-13

**Authors:** Gerardo Cazzato, Anna Colagrande, Antonietta Cimmino, Aurora Demarco, Lucia Lospalluti, Francesca Arezzo, Leonardo Resta, Giuseppe Ingravallo

**Affiliations:** 1 Section of Pathology, Department of Emergency and Organ Transplantation (DETO), University of Bari ‘Aldo Moro’, 70124 Bari, Italy; anna.colagrande@gmail.com (A.C.); micasucci@inwind.it (A.C.); leonardo.resta@uniba.it (L.R.); 2 Section of Dermatology, Department of Biomedical Sciences and Human Oncology, University of Bari ‘Aldo Moro’, 70124 Bari, Italy; aurorademarco94@gmail.com (A.D.); lucia.lospalluti@policlinico.ba.it (L.L.); 3 Section of Gynecologic and Obstetrics Clinic, Department of Biomedical Sciences and Human Oncology, University of Bari ‘Aldo Moro’, 70121 Bari, Italy; francesca.arezzo@uniba.it

**Keywords:** melanoma, pattern, carcinoid-like, paraganglioma-like

## Abstract

Melanoma is among the most aggressive tumors, with different histological patterns of presentation ranging from the usual and easily diagnosable pictures to complex patterns of difficult diagnostic interpretation. Here, we present three cases of a very rare melanoma variant described as “carcinoid-like” and “paraganglioma-like” in the literature, and a brief review of the current literature of the very few cases described to date.

## 1. Introduction

Melanoma is among the most aggressive cancers, with a peak incidence between the ages of 35 and 55, and poses a significant public health problem in terms of morbidity and mortality but also socioeconomic burden [1]. It has long been known that melanoma can have different histological patterns of growth, some but not all of which can be correlated with a worse clinical outcome and biological behavior [2,3]. Among these are more usual patterns such as superficial spreading type melanoma (SSM), lentigo maligna type melanoma (LMM) or nodular melanoma (NM), as well as much rarer patterns in daily clinical practice such as carcinoid-like and paraganglioma-like patterns [3,4]. In this report, we present three cases of very rare variants of melanoma observed in the last 5 years, namely two cases of melanoma with a carcinoid-like pattern and one case of paraganglioma-like melanotic melanoma. Finally, we briefly review the few reported cases of these variants in the literature.

## 2. Materials and Methods

### 2.1. Case 1

A 47-year-old man reported the presence of an irregularly pigmented, asymmetric skin lesion, of 0.7 mm in diameter, on his back that had appeared at an unspecified time before. He did not complain of other concomitant ailments and had no clinical history of significant disease. When the lesion started to bleed spontaneously, he presented to the University Plastic Surgery Complex Operative Unit. After a medical examination, the lesion was removed and the sample was sent to the Pathological Anatomy Department for histological analysis.

### 2.2. Case 2

A 73-year-old man presented to the University Plastic Surgery U.O.C. after finding a brownish lesion, about 1.2 cm in diameter, on the left arm. This lesion was mobile on the underlying planes, not painful nor painful, with slight asymmetry.

### 2.3. Case 3

**Figure 1:**
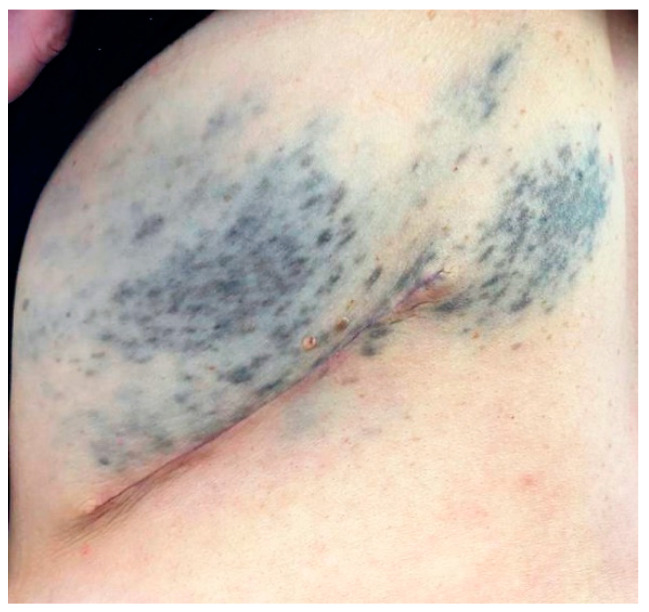
Thoracic melanosis with a characteristic metameric distribution.

A 57-year-old woman presented to Plastic Surgery following the finding of multiple subcutaneous neoformations, present in the thoraco-lumbar region for some time, neither painful nor aching, mobile with respect to the underlying planes. The patient had been affected since birth by thoracic melanosis with a characteristic metameric distribution (Figure 1). Therefore, it was decided to remove 3 of these subcutaneous neoformations and the samples were sent to the Pathological Anatomy laboratory.

Informed consent was obtained from all patients in question. All the samples were fixed in 10% neutral buffered formaldehyde, sampled according to appropriate protocols, dehydrated, embedded in paraffin and microtome cut, obtaining 5 micron-thick sections, stained with Hematoxylin–Eosin and observed under an optical microscope. All the tissues studied were subjected to molecular analysis performed on DNA extracted by Next Generation Sequencing (NGS) using 5 micron-thick tissue sections (FFPE), with >50% of tumor cells present in the sample. The possible presence of somatic variants for the genes reported in Table 1 was tested but in none of the three cases were these somatic variants positive for the panel used. The latter case was also tested for possible mutations for GNAQ, SF3B1 and BAP1, and the results demonstrated the presence of mutation for SF3B1 and BAP1 demonstrated by immunohistochemistry both in the “blue-nevus like” portion and in the melanoma portion.

## 3. Results

**Figure 2:**
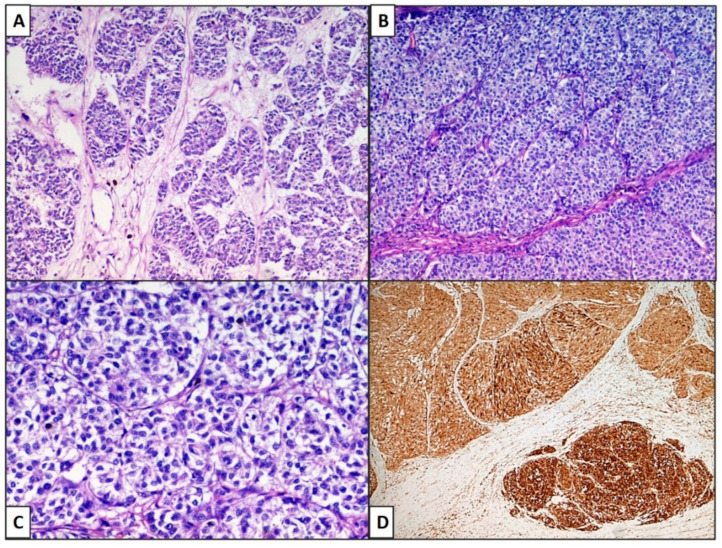
(**A**) Case 1: Neoplastic cells were gathered in very long and intertwined cords, sometimes in elaborated nests or alveolar or insular (organoid) structures. (**B**) Case 2,3: In these cases, there were ribbons, trabeculae and cords of neoplastic cells closely packed together (zellballen pattern). Cytologically, the cells are large, sometimes with pale eosinophilic cytoplasm, but also with clear features (**C**) Case 2: (Hematoxylin–Eosin, Original Magnification 100×). (**D**) Cases 1–3: Melan-A was strongly positive in neoplastic cells. (IHC, original magnification 100×).

The lesions of all three patients showed a very similar pattern: figured, trabecular pattern with nests, alveoli. Neoplastic cells were gathered in very long and intertwined cords, sometimes in elaborate nests or alveolar or insular structures (Figure 2A), constantly surrounded by a thin fibrous capsule and they were usually in a palisade array. In Case 3, there were more ribbons, trabeculae and cords of neoplastic cells, closely packed together (zellballen pattern). Cytologically, the cells are large, sometimes with a pale eosinophilic cytoplasm, but also with clear features (Figure 2C). In some areas, the nuclei were at one pole of the cell, with an irregular nuclear membrane profile and dense chromatin. There was severe necrosis and an elevated mitotic rate. In all three cases, there were extensive deposits of melanic pigment. In the first two cases, a clear junctional neoplastic component was recognized, whereas this characteristic was absent in the third case.

### 3.1. Final Diagnosis

By virtue of the morphological and immunophenotypic picture, a diagnosis was made of melanoma with a prominent carcinoid-like pattern in the first two cases and of a subcutaneous localization of melanoma with a paraganglioma-like pattern in the last case, probably arising from the hemithoracic melanosis which the patient had been affected by since birth. It is important to underline that in the last case, it was not possible to ascertain whether or not the lesion was primitive due to the patient′s characteristic melanosis.

### 3.2. Follow-Up Data

All three patients underwent sentinel lymph node biopsy with different outcomes. In the first two cases the sentinel lymph node was negative, while in the third case there was localization of melanoma in the sentinel lymph node, so this patient underwent removal of a lymph node package, of which 4 lymph nodes were metastatic. This patient is now undergoing immunotherapy treatment with Ipilimumab, a checkpoint inhibitor that targets the CTLA-4 pathway, and at the 6-month follow-up she shows no disease progression.

## 4. Discussion

Melanoma continues to be one of the neoplasms with the highest incidence and the highest mortality rate: the incidence of melanoma in Italy is equal to 5–7 cases per 100,000 inhabitants per year [5,6]. Despite the advent of large-scale and high-resolution genomics, the gold standard for melanoma diagnosis continues to be histopathology, which also remains the primary tool for classification, in conjunction with clinical characteristics [6]. While the more usual patterns are quite typical and few problems are encountered in the diagnosis, several difficulties can arise when faced with rare variants of melanoma [7]. Here, we present three cases of very rare variants of melanoma, in which the neoplastic cells were distributed in carcinoid and paraganglioma patterns. Clinically, these lesions are heterogeneous and can affect patients of all ages, with disparate clinical pictures such as papules (skin tag-like) by Sarma et al. [8] or nodular non-pigmented and painless lesions, as reported by Cimpean et al. [9]. However, no clinical pattern can be reliable in discriminating a PDMT from a melanoma with carcinoid and paraganglioma-like features.

In 2004, Sarma et al. and Deyrup et al. reported histological aspects of eight cases of an entity they called “Paraganglioma-like dermal melanocytic tumor” (PDMT), characterized by ovoid, clear cells, sometimes with an amphophilic cytoplasm and low mitotic activity. The authors described this entity as a benign lesion, albeit potentially liable to progress to mild malignancy [8,10]. All cases reported in this work were positive for melanocyte markers such as S-100 protein, Melan-A and HMB-45, while they were negative for Pan-cytokeratin. In 2009, Cimpean et al. reported a PDMT in a 13-year-old girl, with the same morphological and immunophenotypic characteristics [9].

Similar reports have been received from Thyvalappil et al. [11], Zinovkin et al. [12] and Zhang et al. [13].

All data are summarized in Table 2. The difference between our three cases and these descriptions mainly lies in the high atypia, necrosis and neoplastic proliferation fraction (Ki67+): in all reported cases of PDMT, these three items were absent or very low. It seems correct to say that our three cases are more easily ascribable to melanoma with a carcinoid-like and/or paraganglioma-like pattern. Kacerovska et al. described four cases of melanoma with a carcinoid-like pattern characterized by a distinctive arrangement of the neoplastic cells as trabecules, ribbons, pseudorosettes, rosettes, or small round insular islands, thus closely resembling cell arrangements in carcinoids of various organs [14]. In 2009, Mirzabeigi et al. [15] reported some cases of primary cutaneous melanoma with perivascular pseudorosette characteristics, an unusual morphological pattern added to an unusual positivity for some neuroendocrine markers such as Cromogranin A (CgA) and Neuron Specific Enolase (NSE), both negative in our cases. Finally, in 2013, Ishida et al. [16] reported a case of melanoma with extensive pseudorosette formation, with neoplastic cells that were positive for melanocytic markers. In our opinion, our three cases are to be considered melanomas with a carcinoid-like and paraganglioma-like pattern rather than PDMT. Finally, in relation to the relationship between mucocutaneous melanosis and the development of malignant melanoma, various reports have attempted to address this problem, and it is correct to state that in cases of this thoracic melanosis presenting as a “phakomatosis”, there is a greater risk of developing malignant melanoma [17,18].

## 5. Conclusions

Herein, we describe three new cases of melanoma with unusual and bizarre patterns. Correct knowledge of these entities may help to avoid underestimating these histological variants and making an adequate diagnosis. New histogenetic and molecular studies will be needed to further clarify whether these patterns confer a greater aggressiveness to the neoplastic clone than the more usual histological patterns.

## Figures and Tables

**Table 1:** Genetic panel used for all three patients.

Tested Genes				
ALK	BRAF	EGFR	ERBB2	FGFR3
HRAS	IDH1	ROS1	KIT	KRAS
MET	NRAS	PDGFRA	PIK3CA	RET

**Table 2:** PDMT described in the literature.

Authors	Year	Number of Cases	Gender	Localization
Sarma et al. [8]	2008	1	1 M	Left cheek
Cimpean et al. [9]	2009	1	1 F	Left leg
Deyrup et al. [10]	2004	8	2 M; 6 F	Lower leg (1), Hip (1), Thigh (3), Elbow (2), Knee (1)
Thyvalappil et al. [11]	2015	1	1 M	Right leg
Zinovkin et al. [12]	2015	1	1 M	Right thigh
Zhang et al. [13]	2018	1	1 M	Right neck
